# Systemic immune-inflammation index, neutrophil to high-density lipoprotein ratio and pre-hospital delay: promising biomarkers for predicting the prognosis of patients with acute ST-elevation myocardial infarction

**DOI:** 10.3389/fcvm.2026.1718555

**Published:** 2026-02-03

**Authors:** Pinye Chen, Yiming Wang, Junfang Guo, Tao Rui

**Affiliations:** Division of Cardiology, Department of Medicine, The Affiliated People’s Hospital of Jiangsu University, Zhenjiang, Jiangsu, China

**Keywords:** major adverse cardiovascular event, neutrophil to high-density lipoprotein ratio, pre-hospital delay time, ST-elevation myocardial infarction, systemic immune-inflammation index

## Abstract

**Background:**

Research has confirmed the relationship between acute inflammation, lipid metabolism disorders, and the occurrence of coronary heart disease. However, no studies have analyzed the association between inflammation and lipid-related indicators with the poor prognosis of STEMI (ST-Elevation Myocardial Infarction) patients.

**Methods:**

The retrospective cohort study enrolled 556 patients diagnosed with STEMI. According to 18 Months follow-up outcomes, participants were categorized into either the MACE group or the non MACE group. Additionally, patients were stratified based on their systemic immune-inflammatory index (SII), neutrophil to high-density lipoprotein ratio (NHR), and pre-hospital delay time) levels using optimal cut-off points. The predictive value of SII, NHR and PHDT alone or combined was evaluated using receiver operating characteristic (ROC) curves. Risk factors of MACE in STEMI patients were detected by Multivariable Cox regression analysis. In addition, the Kaplan–Meier approach was applied to assess the long-term survival rates of different. levels of SII, NHR and PHDT in STEMI patient.

**Results:**

The levels of SII, NHR and PHDT were significantly higher in the MACE group as compared to the non-MACE group. The ROC curve analysis demonstrated that SII, NHR and PHDT possess considerable predictive power for the development of MACE in STEMI patients. Furthermore, the area under the curve of the combination of SII, NHR and PHDT was 0.819 (95% CI: 0.78–0.85, *P* < 0.01), which was better than each individual indicator separately. According to the Kaplan–Meier survival curve, patients exhibiting higher SII, NHR and PHDT had an increased incidence of MACE. Univariate and multivariable analyses revealed that SII, NHR and PHDT were independent predictors for the occurrence of MACE.

**Conclusion:**

Elevated levels of SII, NHR and PHDT correlate closely with MACE in patients with STEMI, and demonstrate predictive value.

## 1 Introduction

With the ongoing increase and aging of the global population, cardiovascular disease (CVD) has become a significant challenge to global public health. In 2019, CVD-related mortality was identified as the leading cause of death from non-communicable diseases, accounting for approximately 18.56 million deaths and imposing a substantial health and economic burden on society (1). ST-elevation myocardial infarction (STEMI) is a classic subtype of coronary artery disease, characterized by high mortality and morbidity (2). Although early reperfusion therapy has been widely used in clinical practice, the prognosis of patients with STEMI is far from ideal, and they often suffer from major adverse cardiovascular events (MACE), including heart failure (3), malignant arrhythmias (4), ventricular remodeling (5), and reinfarction. Therefore, other factors affecting the clinical outcomes of STEMI patients need to be further explored. Coronary atherosclerosis (AS) is widely recognized as the pathological basis of STEMI. Studies have demonstrated a significant correlation between the progression of atherosclerosis and the interplay of inflammatory responses (6), lipid metabolism disorders (7), and genetic predispositions (8). Immune cells including neutrophils, lymphocytes, and platelets play key roles in aseptic inflammation in AS (7). Meanwhile, high-density lipoprotein (HDL) has been shown to inhibit the development of AS (9). Consequently, novel biomarkers have been developed based on neutrophils, lymphocytes, platelets, and HDL to evaluate their correlations with CVD resulting from AS. Unlike conventional inflammatory indicators, emerging novel biomarkers encompass a broader spectrum of immune cells and provide a multifaceted assessment of CVD. The Systemic Immune-Inflammation Index (SII) reflects the patients' local immune status and systemic inflammatory burden, comprising neutrophils, lymphocytes, and platelets (10). Elevated SII has been confirmed to serve as a prognostic indicator of adverse events in CVD patients (11), especially in patients with acute myocardial infarction (12). Compared with the Global Registry of Acute Coronary Events (GRACE) scoring system, the multivariable predictive model constructed based on SII showed high predictive values for MACE (13). Neutrophil-to-high-density lipoprotein ratio (NHR) can measure lipid metabolism while reflecting inflammation (14). NHR also possesses predictive values for long-term mortality in older AMI patients (15).

Time is life, and time is the myocardium. Opening the culprit lesion as soon as possible can improve myocardial ischemia to the greatest extent and save the dying myocardium. Although current guidelines emphasize “door-to-balloon time” (16), pre-hospital delay time (PHDT), which refers to the time between the onset of chest pain symptoms and hospital admission, accounts for the majority of total ischemic time and remains a barrier to timely reperfusion (17). Unfortunately, certain patients experience prolonged PHDT due to atypical symptoms (18), advanced age, and underlying diseases (19).

However, current studies have not focused on the relationship of inflammation, lipids, and timing factors with poor prognoses in STEMI patients. Our study aimed to evaluate the correlations of SII, NHR, and PHDT with MACE in STEMI patients and further explored the prognostic value of these indicators, alone and combined for the clinical outcomes of STEMI patients.

## 2 Materials and methods

### 2.1 Study design and participants

**Figure 1:**
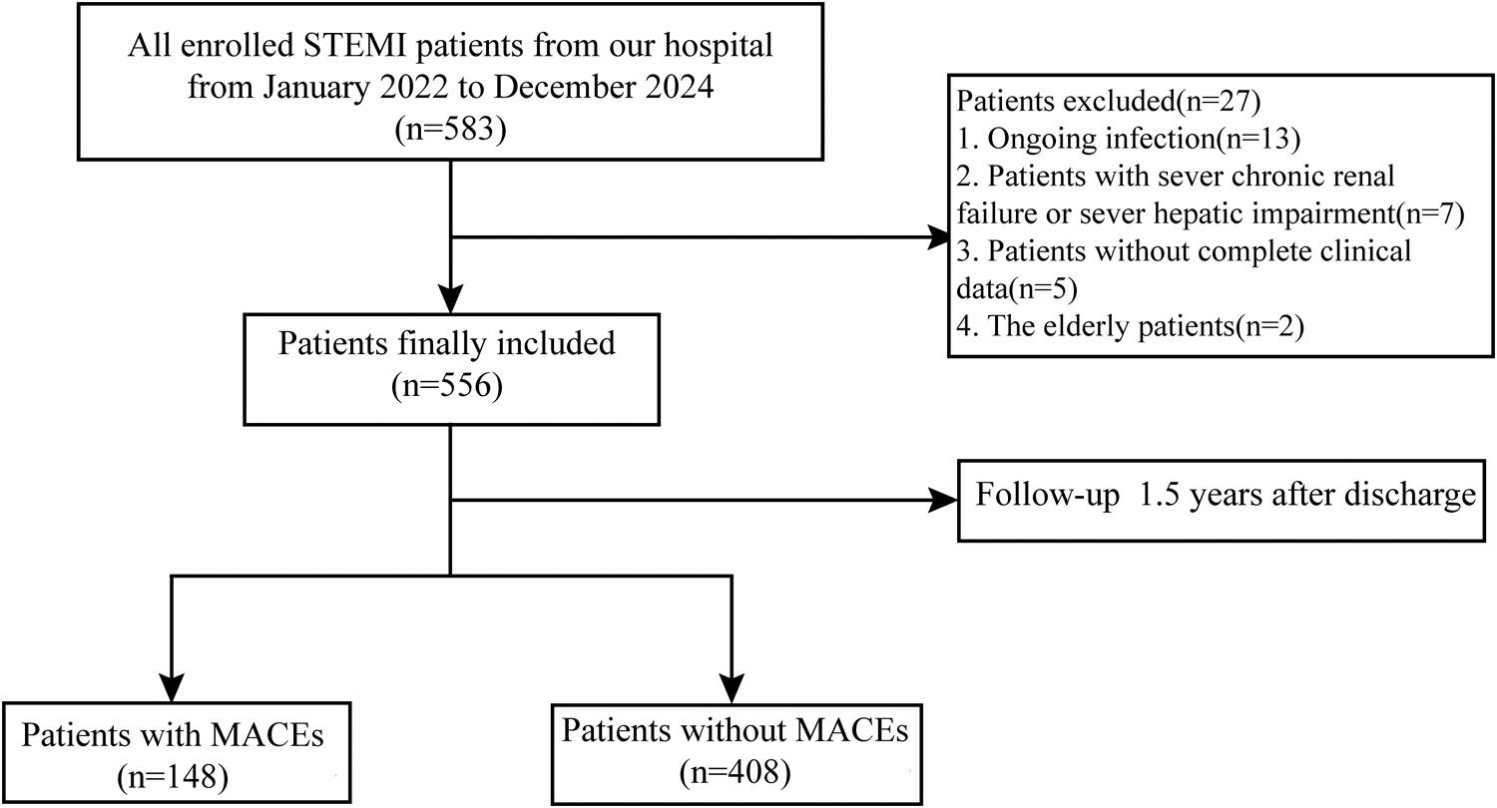
The flow chart of participants inclusion.

This retrospective cohort study included 583 participants who were diagnosed with STEMI at the Affiliated People's Hospital of Jiangsu University from January 2022 to December 2024. The diagnostic criteria for STEMI were outlined as follows: (1) evidence of myocardial injury (elevations of cardiac troponin) with clinical manifestation of myocardial ischemia (20); (2) new ST elevation at the J point in at least 2 contiguous leads (in leads V2-V3, ≥2 mm in men or ≥1.5 mm in women; in other contiguous leads or the limb leads, ≥1 mm) (21). Exclusion criteria were as follows: 1. Older participants (aged ≥90 years); 2. Participants with ongoing infection or autoimmune disease; 3. Participants with severe hepatic or renal impairment, hematological disease, and malignant tumors; 4. Participants without complete clinical data. Following hospital admission, all patients underwent revascularzation therapy via percutaneous coronary intervention. The study initially enrolled 583 participants, and 556 participants were ultimately included according to the exclusion criteria. Details of the study protocol are delineated in Figure 1. Using Receiver operating characteristic (ROC) curves to determine the maximum Youden's Index of SII, NHR and PHDT. The number corresponding to the maximum Youden's Index is the best cut-off point. According to the best cut-off point participants were classified into two groups (Group Q1, SII<892.54 or NHR<6.7 or PHDT<3.75. Group Q2, SII≥892.54, NHR≥6.7, and PHDT≥3.75). There were 437participants in Group Q1 (low SII/low NHR/low PHDT) and 119 participants in Group Q2 (high SII + high NHR + high PHDT).

This study was approved by Affiliated People's Hospital of Jiangsu University Ethics Committee (SQK-20250067-W), and its protocol abides by the principles of the Declaration of Helsinki.

### 2.2 Data collection

Demographic and clinical data were collected by reviewing electronic medical records, including age, sex, past medical history (hypertension, diabetes, myocardial infarction, and percutaneous coronary intervention), smoking and alcohol status, blood routine, lipid, and in-hospital medications. Routine blood test results were acquired immediately after admission. Cardiac imaging was performed by echocardiography and coronary angiography.

The inflammatory indices were calculated as follows: SII = neutrophil count (×10^9^/L) × platelet count (×10^9^/L)/lymphocyte count (×10^9^/L); NHR = neutrophil count (×10^9^/L)/HDL cholesterol (HDL-C) (mmol/L) ratio.

### 2.3 Clinical endpoint

The incidence of MACE during hospitalization or 1.5 years of follow-up after discharge was documented. Follow-up was complete on all participants. MACE were defined as death from all causes, acute heart failure, malignant arrhythmia, unstable angina, and target vessel reconstruction. Any occurrence of the aforementioned events is classified as a MACE event. Information during the follow-up period was collected through standard telephone interviews conduced with patients and their relatives, in additional to the utilization of an electronic medical record system.

### 2.4 Statistical analysis

*SPSS 22.0* and *R 4.3.0* were used for data analyses. Continuous data with a normal distribution were described as mean ± standard deviation, and the two independent samples *t-*test was used for comparisons between the two groups. Continuous data with a skewed distribution were described as [M (Q3–Q1)] and analyzed using the rank sum test for two independent samples. Categorical data were presented as *n* (%) and analyzed using the chi-square test of Fisher's exact probability method. Kaplan–Meier analysis was employed to visualize the incidence of MACE over time across the groups, and the Log-rank test was used. Statistical significance was considered when *P* < 0.05. Cox regression was used to assess the association between included variables and outcome events. Receiver operating characteristic (ROC) curves were plotted to evaluate the predictive value of core indices (SII, NHR, and PHDT), alone or combined. The Delong test was utilized to analyze the predictive value of SII, NHS, and PHDT alone and combined in MACE.

## 3 Results

### 3.1 Comparison of clinical characteristics between the MACE and non-MACE groups

After 1.5 years of follow-up, 148 participants (26.62%) were classified into the MACE group. Among them, 8 participants experienced MACE during hospitalization, while the MACE of remaining 140 participants all occurred during the follow-up period outside the hospital. Compared with the Non-MACE group, participants in the MACE group were older (median age, 67 vs. 59 years; ***P*** < 0.001) and had higher inflammatory markers [neutrophils: 8.0 vs. 6.7; white blood cell count (WBC): 9.9 vs. 9.0; C-reactive protein 7.75 vs. 5.67; SII: 1,147.0 vs. 667; NHR: 8.06 vs. 5.74; both ***P*** ≤ 0.001]. The MACE group also exhibited worse renal function (Cr: 81 vs. 72; ***P*** < 0.001). Left ventricular ultrasonic parameters and ejection fraction differed significantly between groups (all *P* ≤ 0.01). No between-group differences were observed in platelet counts and sex distribution (all *P* > 0.05) (Table 1).

**Table 1:** Comparison of clinical characteristics between the MACE and NMACE group.

Variables	Total (*n* = 556)	Non-MACE group (*n* = 408)	MACE group (*n* = 148)	*P*
Age, M (Q₁, Q₃)	61.00 (52.00, 71.00)	59.00 (51.00, 69.00)	67.00 (55.75, 77.25)	**<** **.** **001**
Gender, *n* (%)				
Male	466 (83.81)	348 (85.29)	118 (79.73)	0.115
Female	90 (16.19)	60 (14.71)	30 (20.27)	
Blood examination				
WBC, M (Q₁, Q₃)	9.30 (8.10, 10.68)	9.00 (7.70, 10.30)	9.90 (8.90, 11.10)	**<** **.** **001**
Neutrophil, M (Q₁, Q₃)	7.29 (5.70, 8.54)	6.70 (5.20, 8.30)	8.00 (7.25, 8.94)	**<** **.** **001**
PLT, M (Q₁, Q₃)	198.00 (163.00, 246.00)	197.00 (163.00, 241.50)	200.00 (163.50, 260.50)	0.205
Lymphocytes, M (Q₁, Q₃)	1.75 (1.38, 2.30)	1.85 (1.50, 2.48)	1.40 (1.08, 1.89)	**<** **.** **001**
CRP, M (Q₁, Q₃)	6.22 (2.47, 13.94)	5.67 (2.29, 10.98)	7.75 (2.65, 21.95)	**0** **.** **010**
HDL-C, M (Q₁, Q₃)	1.11 (0.92, 1.32)	1.17 (0.98, 1.39)	0.99 (0.84, 1.15)	**<** **.** **001**
Cr, M (Q₁, Q₃)	74.00 (62.00, 88.00)	72.00 (61.00, 85.00)	81.00 (66.00, 94.00)	**<** **.** **001**
Troponin, M (Q₁, Q₃)	5.43 (0.11, 15.33)	5.00 (0.06, 13.25)	7.52 (0.32, 24.42)	**0** **.** **002**
Cardiac imaging				
LVDD, M (Q₁, Q₃)	50.00 (47.00, 53.00)	49.00 (46.00, 52.00)	50.00 (47.00, 54.25)	**<** **.** **001**
LVDS, M (Q₁, Q₃)	37.00 (35.00, 40.00)	37.00 (35.00, 40.00)	38.00 (36.00, 43.25)	**<** **.** **001**
LVEF, M (Q₁, Q₃)	47.00 (45.00, 50.00)	48.00 (45.00, 50.00)	46.00 (40.00, 49.00)	**<** **.** **001**
LVSTD, M (Q₁, Q₃)	11.00 (10.00, 12.00)	11.00 (10.00, 12.00)	11.50 (10.00, 12.00)	0.632
LAD, M (Q₁, Q₃)	35.00 (33.00, 37.00)	35.00 (33.00, 37.00)	36.00 (33.75, 39.00)	**<** **.** **001**
Core index				
PHDT, M (Q₁, Q₃)	4.00 (2.00, 7.00)	3.00 (2.00, 5.00)	6.00 (4.00, 15.00)	**<** **.** **001**
NHR, M (Q₁, Q₃)	6.25 (4.69, 8.17)	5.74 (4.00, 7.23)	8.06 (6.72, 9.78)	**<** **.** **001**
SII, M (Q₁, Q₃)	801 (507.91, 1,186.54)	667 (448.35, 1,022.31)	1,147 (926.88, 1,618.45)	**<** **.** **001**

SII, systemic immune-inflammation index; NHR, neutrophil-to-high-density lipoprotein; PDHT, pre-hospital delay time; WBC, white blood cell; PLT, platelet; HDL-C, high density lipoprotein cholesterol; LVDD, left ventricular diastolic dimension; LVDS, left ventricular systolic dimension; LVEF, left ventricular ejection fractions; LAD, left atrium diameter.

Bold values represent statistically significant results (*p* < 0.05).

### 3.2 Comparison of clinical characteristics based on SII, NHR and PHDT best cut-off

The cohort comprised 556 participants (Group Q1: *n* = 437; Group Q2: *n* = 119). Group Q2 demonstrated significantly elevated inflammatory markers (WBC: 10.60 vs. 8.90, *P* < 0.001; neutrophils: 8.50 vs. 6.70, *P* < 0.001)and platelet counts (216.00 vs. 195.00, *P* < 0.001). Compared with group Q2, notable differences were found in cardiac troponin I (7 vs. 5 *P* < 0.001), lymphocyte counts (1.42 vs. 1.83, *P* < 0.001) and HDL-C (1.00 vs. 1.18, *P* < 0.001). Left ventricular ejection fraction was reduced in Group Q2 (48.00% vs. 46.00%, *P* = 0.02). The usage of ACEI/ARB (72.27% vs. 84.21%, *P* < 0.001) was less frequent in Group Q2. No significant differences were observed in the rest of drug administration, sex distribution, or most echocardiographic parameters (*p* > 0.05 for all) (Supplementary Table S1).

### 3.3 ROC curve analysis of SII, NHR and PHDT for MACE

**Figure 2:**
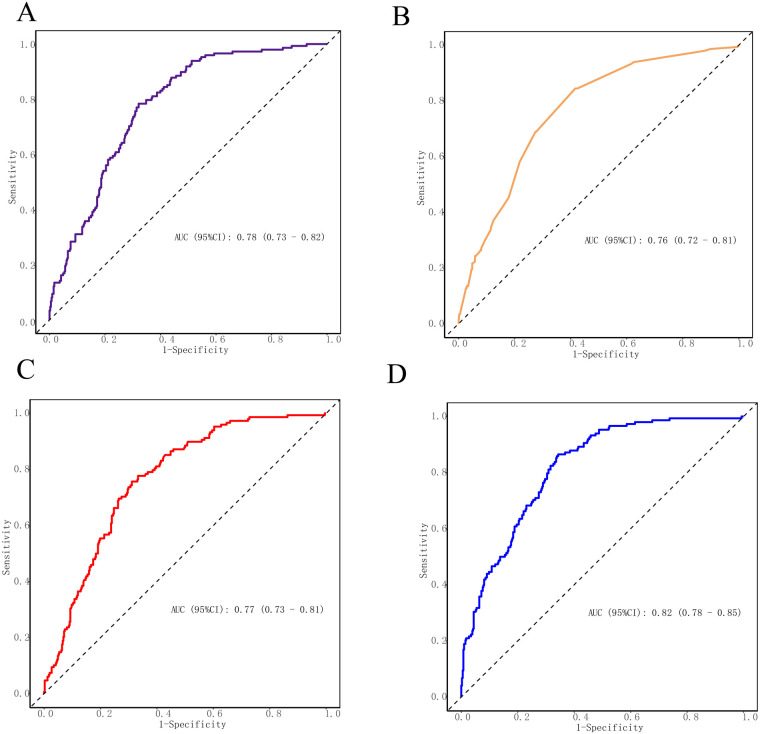
Receiver operating characteristics curves of SII, NHR, PHDT and SII, NHR combined with PHDT for 1.5-year MACE prediction. **(A)** ROC curves were utilized to assess the predictive value of SII for MACE in STEMI patients. **(B)** ROC curves were utilized to assess the predictive value of PHDT for MACE in STEMI patients. **(C)** ROC curves were utilized to assess the predictive value of NHR for MACE in STEMI patients. **(D)** ROC curves were utilized o assess the predictive value of metrics combined for MACE in STEMI patients.

ROC curves were utilized to assess the predictive value of SII, NHR, and PHDT for MACE in STEMI patients. The results showed that the AUCs of SII, PHDT and NHR were 0.78, 0.76, and 0.77, respectively (Figures 2A–C, *P* < 0.001); and the AUC of the combined detection of the three core indicators was 0.82, with a sensitivity of 66% and a specificity of 86% (Table 2, *P* < 0.001). The combination of the three core indicators predicted MACE better than SII, NHR, or PHDT alone (Figure 2D, *P* < 0.001).

**Table 2:** ROC curve analysis for MACE in patients with STEMI.

Indicator	Sensitivity (%)	Specificity (%)	Best cut-off	AUC	95% CI	*P*
SII	0.68	0.78	892.54	0.78	0.73–0.82	0.000
NHR	0.69	0.76	6.70	0.77	0.73–0.81	0.000
PHDT	0.60	0.84	3.75	0.76	0.72–0.81	0.000
Combine	0.66	0.86	0.198	0.82	0.78–0.85	0.000

### 3.4 Risk factors analysis for MACE development

Cox regression was leveraged to filter and evaluate the indicators related to MACE. Under univariate Cox analysis, age, Cr, troponin, Killip, CRP, left ventricular ejection fractions, multivessel disease, SII, NHR and PHDT were selected. Subsequently, indicators with *P* *≤* 0.001 in univariate Cox analysis were included in multivariable Cox analysis. Finally, the results showed that Ln(SII) (HR = 1.73 *P* < 0.001 95% CI 1.30–2.29), Ln(NHR) (HR = 3.25 *P* < 0.001, 95% CI 2.09–5.06), and PHDT (HR = 1.03 *P* < 0.001, 95% CI 1.01–1.04) were significant independent predictor of MACE after adjusting for confounding variables. Age (HR = 1.02 *P* = 0.016 95% CI 1.01–1.03), Troponin (HR = 1.02 *P* = 0.004 95% CI 1.01–1.03), Killip (HR = 2.20 *P* = 0.01 95% CI 1.39–3.51) and multivessel disease (HR = 1.80 *P* = 0.002 95% CI 1.24–2.62) were other independent predictors of MACE (Table 3).

**Table 3:** Univariate and multivariable Cox regression analysis results for MACE.

Variables	Univariate analysis			Multivariable analysis		
	HR	95% CI	*P*-value	HR	95% CI	*P*-value
Age	1.03	1.02–1.05	**<0** **.** **001**	1.02	1.01–1.03	**0** **.** **016**
Cr	1.01	1.01–1.01	**<0** **.** **001**	1.00	1.00–1.00	0.781
Troponin	1.02	1.01–1.03	**<0** **.** **001**	1.02	1.01–1.03	**0** **.** **004**
Killip						
1	Reference			Reference		
2	2.98	1.82–4.89	**<0** **.** **001**	1.12	0.63–2.00	0.708
3	4.55	2.21–9.39	**<0** **.** **001**	2.26	1.01–5.12	0.050
4	4.36	2.88–6.59	**<0** **.** **001**	2.20	1.39–3.51	**<0** **.** **001**
Multivessel disease						
Yes	2.59	1.81–3.70	**<0** **.** **001**	1.80	1.24–2.62	**0** **.** **002**
No	Reference			Reference		
LVEF	0.93	0.91–0.95	**<0** **.** **001**	0.99	0.97–1.02	0.546
LDL-C	0.89	0.72–1.10	0.284			
TC	1.00	0.85–1.16	0.965			
TG	0.93	0.82–1.04	0.201			
CRP	1.01	1.01–1.01	**0** **.** **001**	0.99	0.99–1.00	0.200
PHDT	1.03	1.02–1.04	**<0** **.** **001**	1.03	1.01–1.04	**<0** **.** **001**
Ln(SII)	3.56	2.81–4.51	**<0** **.** **001**	1.73	1.30–2.29	**<0** **.** **001**
Ln(NHR)	5.44	3.83–7.73	**<0** **.** **001**	3.25	2.09–5.06	**<0** **.** **001**

Ln(SII), natural-logarithm of systemic immune-inflammation index; Ln(NHR), natural-logarithm of neutrophil to high-density lipoprotein ratio; LDL-C, low-density lipoprotein cholesterol; TG, triglyceride; TC, serum total cholesterol; CRP, C-reactive protein.

Bold values represent statistically significant results (*p* < 0.05).

### 3.5 Survival analysis

**Figure 3:**
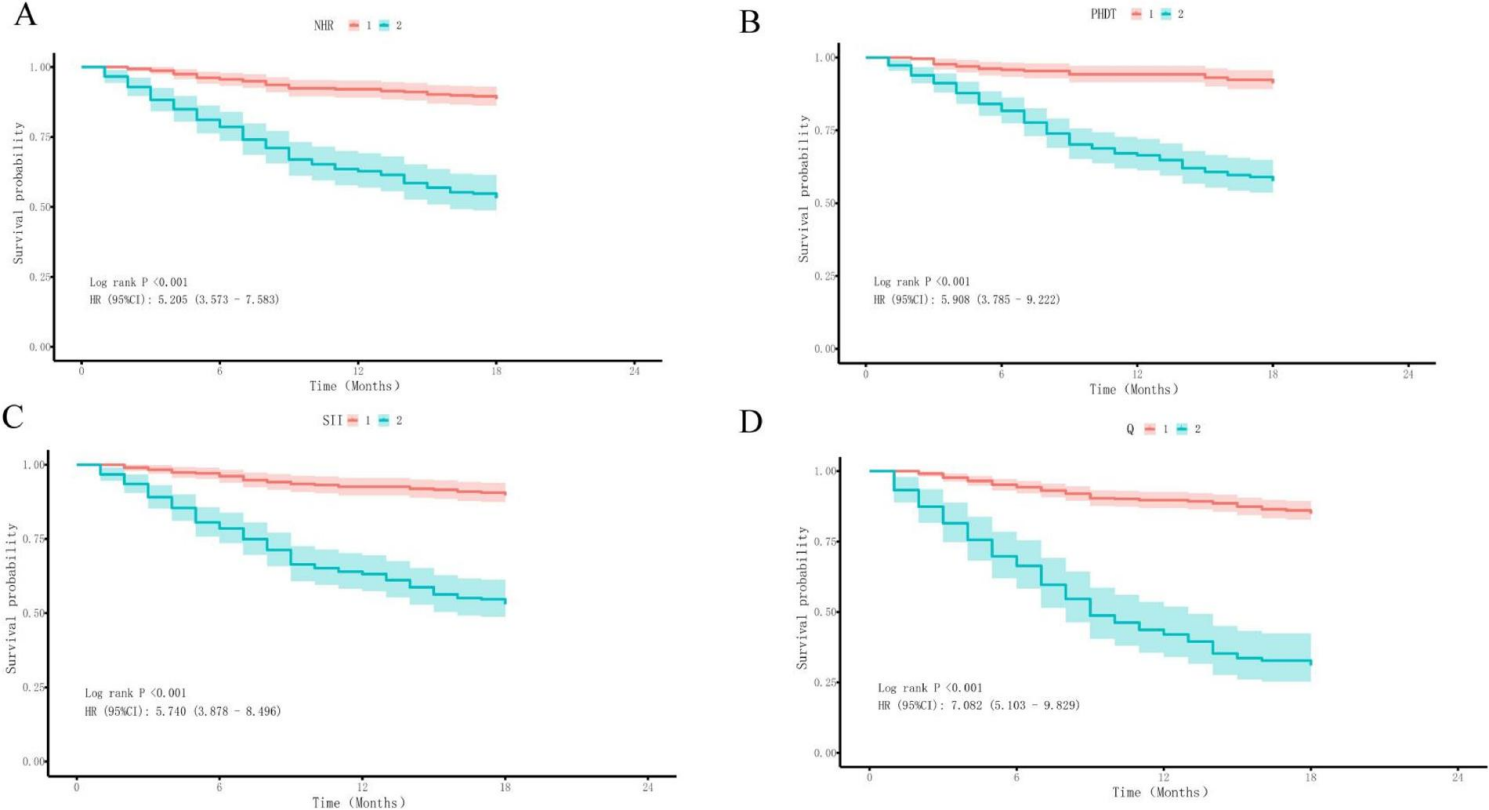
Kaplan–Meier survival curves of MACE in STEMI patients according to the best cut-off of NHR, PHDT, SII. **(A)** Using Kaplan–Meier survival curves to evaluate the incidence of MACE in STEMI patients according to NHR (log rank: 4.897, *P* < 0.001, Group1, NHR<6.7. Group2, NHR≥6.7). **(B)** Using Kaplan–Meier survival curves to evaluate the incidence of MACE in STEMI patients according to PHDT (log rank: 5.325, *P* < 0.001, Group1, PHDT<3.75. Group2, PHDT≥3.75). **(C)** Using Kaplan–Meier survival curves to evaluate the incidence of MACE in STEMI patients according to SII (log rank: 5.688, *P* < 0.001, Group1, SII<892.54. Group2, SII≥892.54). **(D)** Using Kaplan–Meier survival curves to evaluate the incidence of MACE in STEMI patients according to the best cut-off of indices above (log rank: 7.527 *P* < 0.001, GroupQ1, SII<892.54 or NHR<6.7 or PHDT<3.75. GroupQ2, SII≥892.54 and NHR≥6.7 and PHDT≥3.75).

To more intuitively illustrate the correlation between core indicators (SII, NHR, and PHDT) and outcome events, the patients involved were assigned into two groups (Group1 and Group2) according to the best cut-off of those indices. Consequently, Kaplan–Meier survival analysis (Figure 3) was employed to compare the incidence of MACE between the two groups throughout the 1.5-years follow-up. In the group with high SII, high NHR, and high PHDT, MACE incidence was significantly higher (log rank tests: all *P* < 0.001).

## 4 Discussion

This study innovatively integrate inflammatory, lipid, and timing indicators to predict MACE in STEMI patients. The core indexes chosen for this study were derived from common subtypes of WBC and medical history data, characterized by affordability and accessibility. Consequently, SII, NHR, and PHDT are identified as promising biomarkers for MACE in STEMI patients, which might be helpful for clinical practice and treatment.

As novel inflammatory markers based on neutrophils, lymphocytes, platelets, and HDL, the prognostic value of SII and NHR in STEMI patients can be explained by the function of these indicators and the roles they play in the pathophysiology of STEMI patients. From a micro point of view, STEMI is essentially a complex pathological process characterized by immune cell infiltration (22), cytokine activation (23), complement cascade activation (24), and lipid metabolism disorder. Previous studies have highlighted the involvement of multiple immune cells in the inflammatory storm in the early post-myocardial infarction period. Immediately after acute myocardial infarction, polymorphonuclear neutrophils (PMN) are recruited to the infarcted area under the action of high-density chemokines, releasing inflammatory mediators and cytokines (25), thereby aggravating cardiac injury. Regulatory T lymphocytes exert a protective physiological function by secreting anti-inflammatory cytokines (26). Meanwhile, CD4+ helper T cells become activated upon recognition of cardiac autoantigens, thereby facilitating the repair of cardiac wound (27). Studies have shown that STEMI patients with a decreased lymphocyte count are associated with an enhanced risk of recurrence and adverse prognoses (28). Under inflammatory conditions, platelets become activated and interact with immune cells, including WBC and lymphocytes, releasing pro-inflammatory and coagulation factors, further aggravating the local inflammatory response (29). The rupture of atherosclerotic plaque undoubtedly aggravates coronary artery obstruction. HDL plays an antagonistic role in atherosclerosis and is acknowledged as a cardiovascular protective factor (30). Elevated levels of SII and NHR reflect increased counts of peripheral neutrophils and platelets, along with diminished lymphocyte counts and HDL levels, reflecting the pathological process of inflammatory cell activation, immune dysfunction, and lipid metabolism disturbance in STEMI patients. Multiple factors collectively lead to permanent and sustained myocardial injury, which subsequently leads to a decline in myocardial contractility and impairs the prognosis of the patients. According to our study, left ventricular ejection fraction (46% vs. 48%, *P* < 0.01) and troponin levels (7.52 vs. 5, *P* = 0.02) differed significantly between the MACE and non-MACE groups, which further confirms the views above.

Despite the early inflammation storm following AMI, MACE are also closely related to residual inflammatory and lipid risk (31). Residual inflammatory risk (RIR) essentially constitutes a chronic low-grade inflammatory state that is widespread in diseases such as atherosclerosis and AMI. RIR is predominantly attributable to chronic inflammatory cells (PMN, M1 macrophages, and lymphocytes) and inflammatory mediators (32). Meanwhile, residual lipid risk is mediated by LDL-C, triglyceride-rich lipoproteins, and lipoprotein (33). SII and NHR include major biomarkers that determine inflammatory and lipid risk. Compared with conventional inflammatory biomarkers (WBC or CRP), SII and NHR are not only indicative of the proximate CVD risk but also assess the residual cardiovascular risk. Consequently, those novel biomarkers could more accurately reflect the influence of dynamic evolution of inflammatory and lipid burden on patients' prognosis. The above evidence enhances the depth and robustness of our research fundings, as well as augmenting the persuasive validity of our study.

Based on previous clinical experience, older patients typically have weaker inflammatory responses and lower inflammatory cell counts. Interestingly, our study showed higher levels of inflammatory markers in older patients with STEMI, contrary to conventional clinical observations. Relevant studies have stated that older patients exhibit worse immunological competence because of the decline of the adaptive and innate immune system (34). Immune dysfunction can lead to long- term chronic inflammation, characterized by elevated inflammatory markers in the circulating blood (35). This phenomenon is known as “inflamm-aging” (35), and may partly explain the higher inflammatory markers observed in older STEMI patients.

The GRACE score is a widely used clinical risk assessment tool for evaluating the severity of acute coronary syndrome (ACS) and directing medical interventions (36). It is well acknowledged that the GRACE score can predict in-hospital risk and long-term adverse outcomes in patients with ACS (37). In our study, we incorporated the primary indicators from the GRACE score (age, Cr, Killip, troponin), in conjunction with the core indicators (SII, NHR and PHDT), into the COX regression model for comparative analysis. The findings revealed that the SII and NHR exhibited more pronounced prognostic predictive value. Meanwhile, A recent study developed a prognostic model based on a multitude of clinical, biochemical, and imaging markers, with the SII serving as the pivotal element. Statistical analysis revealed that the predictive value of this model was greater than that of the GRACE score system (13). To a certain degree, this highlights the potential limitations of GRACE scores, which ignore the influence of factors like inflammation and lipid on diseases. Therefore, a multi-center cohort study with a larger sample size should be conducted to construct a prognostic model with inflammation and other indicators, thereby providing more guidance for the clinical management of ACS patients.

In conclusion, our study further expanded the scope of prior research. The relationship of inflammation, lipid, and time factors with MACE in STEMI patients was investigated. The findings indicated that SII, NHR, and PHDT were independently associated with the onset and progression of MACE, and the combination of them can effectively predict MACE. However, our research also has certain limitations: 1. The present study was a single-center retrospective cohort study with a small size, which may have introduced selection bias. 2. SII and NHR were not measured repeatedly. As a result, the dynamic changes in core indicators could not be observed and statistically analyzed. 3. This study did not include non-STEMI and other AMI patients, which limited the generalizability of the conclusion.

## 5 Conclusion

Our study demonstrates that high levels of SII, NSH, and PHDT in STEMI patients are positively correlated with an increased risk of MACE. A combination of SII, NHR, and PHDT shows favorable predictive value for MACE in STEMI patients. These findings might be useful for constructing a powerful prognostic model for identifying STEMI patients at high risk of MACE. Furthermore, a theoretical foundation is established for future combined therapeutic strategies targeting the reduction of lipid and inflammatory burdens in patients with STEMI, with the objective of improving clinical outcomes in this population.

## Data Availability

The raw data supporting the conclusions of this article will be made available by the authors, without undue reservation.

## References

[1] Bai J Cui J Shi F Yu C. Global epidemiological patterns in the burden of main non-communicable diseases, 1990–2019: relationships with socio-demographic Index. Int J Public Health. (2023) 68:1605502. 10.3389/ijph.2023.1605502 36726528 PMC9884670

[2] Bajaj A Sethi A Rathor P Suppogun N Sethi A. Acute complications of myocardial infarction in the current era: diagnosis and management. J Invest Med. (2015) 63(7):844–55. 10.1097/JIM.0000000000000232 26295381

[3] Harrington J Jones WS Udell AJ Hannan K Bhatt DL Anker DS Acute decompensated heart failure in the setting of acute coronary syndrome. *JACC Heart Fail*. (2022) 10(6):404–14. 10.1016/j.jchf.2022.02.008 35654525

[4] Garcia R. Ventricular fibrillation in acute myocardial infarction: 20-year trends in the FAST-MI study. Eur Heart J. (2022) 43(47):4887–96. 10.1093/eurheartj/ehac579 36303402

[5] Kramer DG Trikalinos TA Kent DM Antonopoulos GV Konstam MA Udelson JE. Quantitative evaluation of drug or device effects on ventricular remodeling as predictors of therapeutic effects on mortality in patients with heart failure and reduced ejection fraction: a meta-analytic approach. *J Am Coll Cardiol.* (2010) 56(5):392–406. 10.1016/j.jacc.2010.05.011 20650361 PMC4523221

[6] Wolf D Ley K. Immunity and inflammation in atherosclerosis. Circ Res. (2019) 124(2):315–27. 10.1161/CIRCRESAHA.118.313591 30653442 PMC6342482

[7] Schaftenaar F Frodermann V Kuiper J Lutgens E. Atherosclerosis: the interplay between lipids and immune cells. Curr Opin Lipidol. (2016) 27(3):209–15. 10.1097/MOL.0000000000000302 27031276

[8] Kobiyama K Ley K. Atherosclerosis. Circ Res. (2018) 123(10):1118–20. 10.1161/CIRCRESAHA.118.313816 30359201 PMC6298754

[9] Choi HY Iatan I Ruel I Brown L Hales L Choi S Docetaxel as a model compound to promote HDL (high-density lipoprotein) biogenesis and reduce atherosclerosis. Arterioscler Thromb Vasc Biol. (2023) 43(5):609–17. 10.1161/ATVBAHA.122.318275 36861478

[10] Hu B Yang XR Xu Y Sun YF Sun C Guo W Systemic immune-inflammation Index predicts prognosis of patients after curative resection for hepatocellular carcinoma. Clin Cancer Res. (2014) 20(23):6212–22. 10.1158/1078-0432.CCR-14-0442 25271081

[11] Zhao Z Zhang X Sun T Huang X Ma M Yang S Prognostic value of systemic immune-inflammation index in CAD patients: systematic review and meta-analyses. Eur J Clin Investig. (2024) 54(2):e14100. 10.1111/eci.14100 37776036

[12] Zhu Y He H Qiu H Shen G Wang Z Li W. Prognostic value of systemic immune-inflammation Index and NT-proBNP in patients with acute ST-elevation myocardial infarction. Clin Interv Aging. (2023) 18:397–407. 10.2147/CIA.S397614 36959838 PMC10029373

[13] Li X Yu C Liu X Chen Y Wang Y Liang H A prediction model based on systemic immune-inflammatory Index combined with other predictors for Major adverse cardiovascular events in acute myocardial infarction patients. J Inflamm Res. (2024) 17:1211–25. 10.2147/JIR.S443153 38410422 PMC10895983

[14] Ren H Zhu B Zhao Z Li Y Deng G Wang Z Neutrophil to high-density lipoprotein cholesterol ratio as the risk mark in patients with type 2 diabetes combined with acute coronary syndrome: a cross-sectional study. Sci Rep. (2023) 13(1):7836. 10.1038/s41598-023-35050-6 37188740 PMC10185574

[15] Huang JB Chen YS Ji HY Xie WM Jiang J Ran LS Neutrophil to high-density lipoprotein ratio has a superior prognostic value in elderly patients with acute myocardial infarction: a comparison study. Lipids Health Dis. (2020) 19(1):59. 10.1186/s12944-020-01238-2 32247314 PMC7126405

[16] Rao SV O’donoghue ML Ruel M Rab T Tamisholland JE Alexander JH 2025 ACC/AHA/ACEP/NAEMSP/SCAI guideline for the management of patients with acute coronary syndromes: a report of the American College of Cardiology/American Heart Association joint committee on ClinicalPractice. Guidelines. Circulation. (2025) 151(13):CIR.0000000000001309. 10.1161/CIR.0000000000001309 40163565

[17] Ängerud KH Sederholm Lawesson S Isaksson RM Thylen Y Swahn E. Differences in symptoms, first medical contact and pre-hospital delay times between patients with ST- and non-STelevation myocardial infarction. Eur Heart J Acute Cardiovasc Care. (2019) 8(3):201–7. 10.1177/2048872617741734 29111768

[18] Fanta K Daba FB Asefa ET Melaku T Chelkeba L Fekadu G Management and 30-day mortality of acute coronary syndrome in a resource-limited setting: insight from Ethiopia. A prospective cohort study. Front Cardiovasc Med. (2021) 8:707700. 10.3389/fcvm.2021.707700 34604351 PMC8484752

[19] Ting HH Bradley EH Wang Y Lichtman JH Nallamothu BK Sullivan MD Factors associated with Longer time from symptom onset to hospital presentation for patients with ST-elevation myocardial infarction. Arch Intern Med. (2008) 168(9):959. 10.1001/archinte.168.9.959 18474760 PMC4858313

[20] Ibanez B James S Agewall S Antunes MJ Bucciarelli-Ducci C Bueno H 2017 ESC guidelines for the management of acute myocardial infarction in patients presenting with ST-segment elevation. Eur Heart J. (2018) 39(2):119–77. 10.1093/eurheartj/ehx393 28886621

[21] Frampton J Devries JT Welch TD Gersh BJ. Modern management of ST-segment elevation myocardial infarction. Curr Probl Cardiol. (2020) 45(3):100393. 10.1016/j.cpcardiol.2018.08.005 30660333

[22] Ong SB Hernández-Reséndiz S Crespo-Avilan GE Mukhametshina RT Wek XY Cabrera-Fuentes HA Inflammation following acute myocardial infarction: multiple players, dynamic roles, and novel therapeutic opportunities. Pharmacol Ther. (2018) 186:73–87. 10.1016/j.pharmthera.2018.01.001 29330085 PMC5981007

[23] Fanola CL Morrow DA Cannon CP Jarolm P Lukas MA Bode C Interleukin-6 and the risk of adverse outcomes in patients after an acute coronary syndrome: observations from the SOLID-TIMI 52 (stabilization of plaque using darapladib—thrombolysis in myocardial infarction 52) trial. J Am Heart Assoc. (2017) 6(10):e005637. 10.1161/JAHA.117.005637 29066436 PMC5721825

[24] Orrem HL Nilsson PH Pischke SE Kleveland O Yndestad A Ekholt K IL-6 Receptor inhibition by tocilizumab attenuated expression of C5a receptor 1 and 2 in non-ST-elevation myocardial infarction. Front Immunol. (2018) 9:2035. 10.3389/fimmu.2018.02035 30258440 PMC6143659

[25] Maier W Altwegg LA Corti R Gay S Hersberger M Maly FE Inflammatory markers at the site of ruptured plaque in acute myocardial infarction: locally increased interleukin-6 and Serum amyloid A but decreased C-reactive protein. Circulation. (2005) 111(11):1355–61. 10.1161/01.CIR.0000158479.58589.0A 15753219

[26] Meng X Yang J Dong M Drummond GR. Regulatory T cells in cardiovascular diseases. Nat Rev Cardiol. (2016) 13(3):167–79. 10.1038/nrcardio.2015.169 26525543 PMC11849084

[27] Hofmann U Beyersdorf N Weirather J Podolskaya A Bauersachs J Ertl G Activation of CD4+ T lymphocytes improves wound healing and survival after experimental myocardial infarction in mice. Circulation. (2012) 125(13):1652–63. 10.1161/CIRCULATIONAHA.111.044164 22388323

[28] Núñez J Núñez E Bodí V Sanchis J Mainar L Minana G Low lymphocyte count in acute phase of ST-segment elevation myocardial infarction predicts long-term recurrent myocardial infarction. Coron Artery Dis. (2010) 21(1):1–7. 10.1097/MCA.0b013e328332ee15 20050312

[29] Schrottmaier WC Mussbacher M Salzmann M Assinger A. Platelet-leukocyte interplay during vascular disease. Atherosclerosis. (2020) 307:109–20. 10.1016/j.atherosclerosis.2020.04.018 32439204

[30] Song Y Yang Y Zhang J Wang W He W Zhang X The apoB100/apoAI ratio is independently associated with the severity of coronary heart disease: a cross sectional study in patients undergoing coronaryangiography. Lipids Health Dis. (2015) 14(1):150. 10.1186/s12944-015-0155-6 26582246 PMC4652387

[31] Gomez-Delgado F Raya-Cruz M Katsiki N Delgado-Lista J Perez-Martinez P. Residual cardiovascular risk: when should we treat it? Eur J Intern Med. (2024) 120:17–24. 10.1016/j.ejim.2023.10.013 37845117

[32] Matter MA Paneni F Libby P Frantz S Stahil BE Templin C Inflammation in acute myocardial infarction: the good, the bad and the ugly. Eur Heart J. (2024) 45(2):89–103. 10.1093/eurheartj/ehad486 37587550 PMC10771378

[33] Hoogeveen RC Ballantyne CM. Residual cardiovascular risk at low LDL: remnants, lipoprotein(a), and inflammation. Clin Chem. (2021) 67(1):143–53. 10.1093/clinchem/hvaa252 33257928 PMC7793228

[34] Frasca D Blomberg BB. Inflammaging decreases adaptive and innate immune responses in mice and humans. Biogerontology. (2016) 17(1):7–19. 10.1007/s10522-015-9578-8 25921609 PMC4626429

[35] Ferrucci L Fabbri E. Inflammageing: chronic inflammation in ageing, cardiovascular disease, and frailty. Nat Rev Cardiol. (2018) 15(9):505–22. 10.1038/s41569-018-0064-2 30065258 PMC6146930

[36] Moledina SM Kontopantelis E Wijeysundera HC Frantz S Stahil BE Templin C Ethnicity-dependent performance of the global registry of acute coronary events risk score for prediction of non-ST-segment elevation myocardial infarction in-hospital mortality: nationwide cohort study. Eur Heart J. (2022) 43(24):2289–99. 10.1093/eurheartj/ehac052 35202472

[37] Meune C Drexler B Haaf P Reichlin T Reiter M Meissner J The GRACE score’s performance in predicting in-hospital and 1-year outcome in the era of high-sensitivity cardiac troponin assays and btype natriuretic peptide. Heart. (2011) 97(18):1479–83. 10.1136/hrt.2010.2209 21444339

