# Supplementary material for: Systemic immune-inflammation index, neutrophil to high-density lipoprotein ratio and pre-hospital delay: promising biomarkers for predicting the prognosis of patients with acute ST-elevation myocardial infarction
Source: Front Cardiovasc Med. 2026 Feb 3;13:1718555. doi: 10.3389/fcvm.2026.1718555 (PMC12909210; doi:10.3389/fcvm.2026.1718555)
Supplement: Supplementary file 1 [file Table1.docx]

Supplementary Material

Supplementary Table 1 Comparison of clinical characteristics based on SII, NHR and PHDT best cut-off

| **Variables** | **Low SII/Low NHR/Low PHDT**  **（GroupQ1 n = 437)** | **High SII+ High NHR+ High PHDT**  **（GroupQ2 n = 119)** | ***P*** |
| --- | --- | --- | --- |
|  |  |  |  |
| Age, M (Q₁, Q₃) | 60.00 (52.00, 70.00) | 65.00 (53.00, 73.50) | 0.062 |
| PHDT, M (Q₁, Q₃) | 3.00 (2.00, 5.00) | 7.00 (5.00, 16.00) | **<.001** |
| WBC, M (Q₁, Q₃) | 8.90 (7.69, 10.00) | 10.60 (9.46, 12.00) | **<.001** |
| N, M (Q₁, Q₃) | 6.70 (5.20, 8.10) | 8.50 (7.80, 9.80) | **<.001** |
| PLT, M (Q₁, Q₃) | 195.00 (159.00, 238.00) | 216.00 (182.50, 267.50) | **<.001** |
| L, M (Q₁, Q₃) | 1.83 (1.44, 2.40) | 1.42 (1.09, 1.80) | **<.001** |
| CRP, M (Q₁, Q₃) | 5.86 (2.48, 11.76) | 7.69 (2.46, 25.36) | 0.072 |
| NHR, M (Q₁, Q₃) | 5.70 (4.08, 7.02) | 8.57 (7.57, 10.87) | **<.001** |
| SII, M (Q₁, Q₃) | 679.56 (452.57, 999.77) | 1325.45 (1059.33, 1713.44) | **<.001** |
| HDLC, M (Q₁, Q₃) | 1.18 (0.96, 1.39) | 1.00 (0.83, 1.10) | **<.001** |
| LDLC, M (Q₁, Q₃) | 2.60 (2.15, 3.13) | 2.50 (2.04, 3.12) | 0.166 |
| Cr, M (Q₁, Q₃) | 73.00 (62.00, 86.00) | 77.00 (63.00, 94.00) | 0.076 |
| TC, M (Q₁, Q₃) | 4.42 (3.86, 5.17) | 4.18 (3.62, 5.11) | 0.066 |
| TG, M (Q₁, Q₃) | 1.55 (1.04, 2.30) | 1.42 (1.02, 2.34) | 0.560 |
| Troponin, M (Q₁, Q₃) | 5.00 (0.05, 14.20) | 7.00 (0.67, 19.30) | **0.032** |
| LVDD, M (Q₁, Q₃) | 50.00 (47.00, 52.00) | 50.00 (46.50, 54.00) | 0.483 |
| LVDS, M (Q₁, Q₃) | 37.00 (35.00, 40.00) | 38.00 (35.00, 42.00) | 0.202 |
| LVEF, M (Q₁, Q₃) | 48.00 (45.00, 50.00) | 46.00 (42.00, 49.00) | **0.002** |
| LVSTD, M (Q₁, Q₃) | 11.00 (10.00, 12.00) | 12.00 (10.00, 12.00) | 0.439 |
| LVPWTD, M (Q₁, Q₃) | 9.00 (8.00, 9.00) | 9.00 (8.00, 9.00) | 0.987 |
| AORD, M (Q₁, Q₃) | 34.00 (32.00, 36.00) | 34.00 (31.00, 36.00) | 0.312 |
| LAD, M (Q₁, Q₃) | 35.00 (33.00, 37.00) | 35.00 (33.00, 38.00) | 0.066 |
| FS, M (Q₁, Q₃) | 24.00 (22.00, 25.00) | 23.00 (21.00, 25.00) | **0.007** |
| MACEs, n (%) | 66 (15.10) | 82 (68.91) | **<.001** |
| Gender, n (%) |  |  | 0.836 |
| Male | 367 (83.98) | 99 (83.19) |  |
| Female | 70 (16.02) | 20 (16.81) |  |
| Smoking, n (%) | 255 (58.35) | 70 (58.82) | 0.926 |
| Alcohol, n (%) | 90 (20.59) | 29 (24.37) | 0.373 |
| Hypertension, n (%) | 239 (54.69) | 69 (57.98) | 0.522 |
| Diabetes mellitus, n (%) | 129 (29.52) | 48 (40.34) | **0.025** |
| Betablockers, n (%) | 368 (84.21) | 86 (72.27) | **0.003** |
| ACEIARB, n (%) | 375 (85.81) | 85 (71.43) | **<.001** |
| CCB, n (%) | 16 (3.66) | 2 (1.68) | 0.429 |
| Stain, n (%) | 437 (100.00) | 118 (99.16) | 0.214 |
| Insulin, n (%) | 27 (6.18) | 8 (6.72) | 0.828 |
| Aspirin, n (%) | 437 (100.00) | 118 (99.16) | 0.214 |
| Clopidogrel, n (%) | 437 (100.00) | 118 (99.16) | 0.214 |
| History PCI, n (%) | 24 (5.49) | 12 (10.08) | 0.071 |
| History MI, n (%) | 24 (5.49) | 12 (10.08) | 0.071 |
| Culprit lesion, n (%) |  |  | 0.384 |
| LM | 9 (2.06) | 2 (1.68) |  |
| LAD | 264 (60.41) | 62 (52.10) |  |
| RCA | 134 (30.66) | 44 (36.97) |  |
| LCX | 30 (6.86) | 11 (9.24) |  |
| Multivessel disease, n (%) | 216 (49.43) | 75 (63.03) | **0.008** |
| TIMI, n( %) |  |  | **0.046** |
| 0 | 0 (0.00) | 1 (0.84) |  |
| 1 | 0 (0.00) | 1 (0.84) |  |
| 2 | 0 (0.00) | 0 (0.00) |  |
| 3 | 437 (100.00) | 117 (98.32) |  |
| Killip, n (%) |  |  | **0.010** |
| 1 | 370 (84.67) | 85 (71.43) |  |
| 2 | 27 (6.18) | 12 (10.08) |  |
| 3 | 8 (1.83) | 4 (3.36) |  |
| 4 | 32 (7.32) | 18 (15.13) |  |

**Abbreviations:** WBC, white blood cell; PLT, platelet; HDL-C, high density lipoprotein cholesterol; LDL-C, low density lipoprotein cholesterol; ACEI, angiotensin converting enzyme inhibitor; ARB, angiotensin receptor inhibitor; CCB, calcium channel blockers; LVDD, left ventricular diastolic dimension; LVDS, left ventricular systolic dimension; LVEF, left ventricular ejection fractions; LVSTD, interventricular septum; LVPWTD, left ventricular posterior wall thickness; AORD, aortic root diameter; LAD, left atrium diameter; LM, left main coronary artery; RCA, right coronary artery; LCX, left circumflex artery
